# Early Evaluation of Patients on Axial Flow Pump Support for Refractory Cardiogenic Shock Is Associated with Left Ventricular Recovery

**DOI:** 10.3390/jcm9124130

**Published:** 2020-12-21

**Authors:** Jérôme Fagot, Frédéric Bouisset, Laurent Bonello, Caroline Biendel, Thibaut Lhermusier, Jean Porterie, Jerome Roncalli, Michel Galinier, Meyer Elbaz, Olivier Lairez, Clément Delmas

**Affiliations:** 1 Intensive Cardiac Care Unit, Department of Cardiology, Rangueil University Hospital, 31059 Toulouse, France; jerome.f-c@hotmail.fr (J.F.); bouisset.f@chu-toulouse.fr (F.B.); biendel.c@chu-toulouse.fr (C.B.); lhermusier.t@chu-toulouse.fr (T.L.); roncalli.j@chu-toulouse.fr (J.R.); galinier.m@chu-toulouse.fr (M.G.); elbaz.m@chu-toulouse.fr (M.E.); lairez.o@chu-toulouse.fr (O.L.); 2 Cardiac Imaging Center, University Hospital of Toulouse, 31059 Toulouse, France; 3 Medical School, Toulouse III Paul Sabatier University, 31059 Toulouse, France; laurent.bonello@ap-hm.fr; 4 Intensive Care Unit, Department of Cardiology, Centre Hospitalo-Universitaire Nord, Aix-Marseille Univeristy, 13385 Marseille, France; 5 Association for Research and Studies in Cardiology (MARS Cardio), 13015 Marseille, France; 6 Department of Cardiovascular Surgery, Rangueil University Hospital, 31059 Toulouse, France; porterie.j@chu-toulouse.fr

**Keywords:** cardiogenic shock, percutaneous left ventricle assist device, Impella^®^, mortality

## Abstract

We investigated prognostic factors associated with refractory left ventricle (LV) failure leading to LV assist device (LVAD), heart transplant or death in patients on an axial flow pump support for cardiogenic shock (CS). Sixty-two CS patients with an Impella^®^ CP or 5.0 implant were retrospectively enrolled, and clinical, biological, echocardiographic, coronarographic and management data were collected. They were compared according to the 30-day outcome. Patients were mainly male (*n* = 55, 89%), 58 ± 11 years old and most had no history of heart failure or coronary artery disease (70%). The main etiology of CS was acute coronary syndrome (*n* = 57, 92%). They presented with severe LV failure (LV ejection fraction (LVEF) 22 ± 9%), organ malperfusion (lactate 3.1 ± 2.1 mmol/L), and frequent use of inotropes, vasopressors, and mechanical ventilation (59, 66 and 30%, respectively). At 24 h, non-recovery was associated with higher total bilirubin (odds ratios (OR) 1.07 (1.00–1.14); *p* = 0.039), lower LVEF (OR 0.89 (0.81–0.96); *p* = 0.006) and the number of administrated amines (OR 4.31 (1.30–14.30); *p* = 0.016). Early evaluation in patients with CS with an axial flow pump implant may enable the identification of factors associated with an unlikely recovery and would call for early screening for LVAD or heart transplant.

## 1. Introduction

Cardiogenic shock (CS), the most severe form of acute heart failure, is a life-threatening condition resulting in low cardiac output, which causes hypotension, hypoxia, organ malperfusion and systemic inflammatory response syndrome [1]. Acute myocardial infarction (AMI) with left ventricle (LV) systolic failure is the main cause of CS, and accounts for approximately 40 to 60% of the cases of CS [2,3], with 5 to 15% of AMIs evolving towards CS (AMICS) [4,5].

Despite improvements in CS management over the past few decades, especially in culprit lesion revascularization in AMI patients [6], recent registries have shown a decreased incidence of AMICS [7] but mortality rates remain extremely high, reaching 35 to 50% [4,5,6,7,8]. This trend in mortality may be partly explained by the lack of evidence in the field of CS management with few large-scale studies. Consequently, most of the available recommendations regarding therapeutic strategies are based on expert consensus. Both the European Society of Cardiology and the American Heart Association recommend specific treatment (especially early revascularization in AMICS), inotropes and/or vasopressors, along with diuretics or a fluid challenge according to the patient’s fluid volume status, as first line therapies for CS. In refractory CS, with persistent hemodynamic instability or progressive multi-organ failure, these recommendations state that mechanical circulatory support (MCS) may be considered to improve hemodynamics, coronary and systemic perfusion, gas exchange and end-organ function [9,10]. Therefore, these devices may allow patients to survive with partial or complete myocardial recovery without need for a long-term LV assist device (LVAD) or a heart transplant. Otherwise, MCS should stabilize myocardial and organ functions, enabling a complete patient evaluation and heart team discussion of the initiation/rejection of a long-term curative plan for end-stage heart failure.

Among the MCS devices is the Impella^®^ device, an axial-flow pump (AFP) placed between the left ventricle and the ascending aorta through the aortic valve, with several pump sizes delivering maximal flows of 2.5 to 5.0 L/min, implanted by a percutaneous, catheter-based approach (Impella^®^ 2.5 or 3.5) or by surgical cannulation (Impella^®^ 5.0).

Nonetheless, while there are an increasing amount of observational data regarding the use of AFP in CS, evidence to establish its impact on patient outcome, and to what extent and when it should be included in the CS management algorithm, is still greatly lacking. Furthermore, recent registry studies have advised caution regarding the safety of Impella^®^ use in CS suggesting increased bleeding risks compared to intra-aortic balloon pump therapy [11,12].

Our aim was to describe the characteristics of patients with CS with an Impella^®^ device in our center, and to identify prognostic factors associated with refractory LV failure leading to LVAD, heart transplant or death.

## 2. Experimental Section

**Figure 1:**
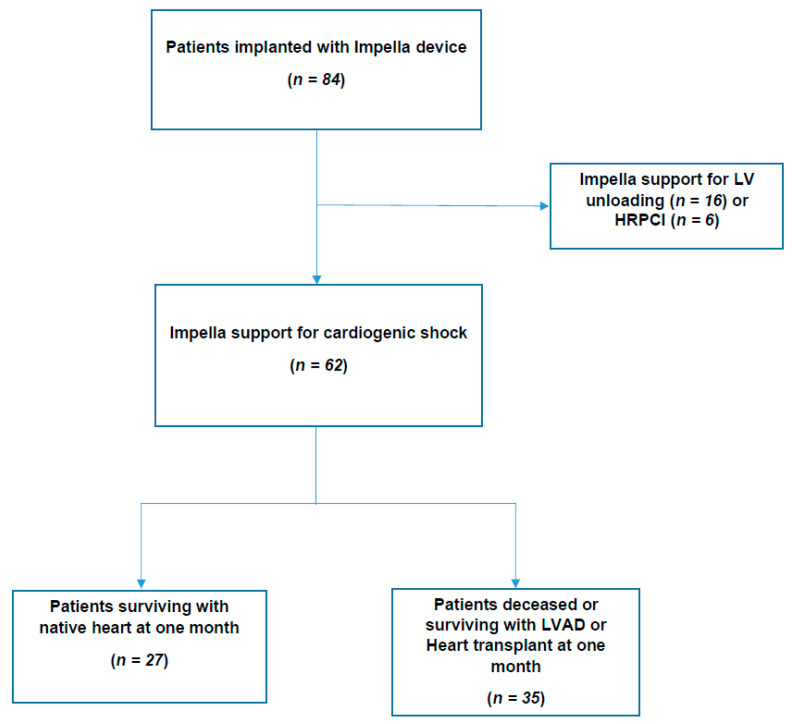
Flowchart; LV: left ventricle; LVAD: left ventricle assist device; PCI: percutaneous coronary intervention; VA-ECMO: venoarterial extracorporeal membrane oxygenation.

All consecutive CS patients supported with an Impella^®^ device (CP or 5.0) (Abiomed, Berlin, Germany) at Toulouse University Hospital were retrospectively included between January 2011 and January 2020 (Figure 1). 

Cardiogenic shock was defined by a combination of 3 criteria, according to the FRENSHOCK registry [3]: (1) low cardiac output defined as a low cardiac index < 2.2 L/min/m^2^ and/or a systolic blood pressure (SBP) < 90 mmHg or vasopressors/inotropes needed to maintain a SBP > 90 mmHg; (2) right and/or left ventricular overload defined by clinical signs, natriuretic peptide elevation, radiological or echocardiographic signs and/or right heart catheterization; (3) organ malperfusion defined by clinical signs (oliguria, confusion, pale and/or cold extremities, mottled skin) and/or biological signs (lactate > 2 mmol/L, metabolic acidosis, renal failure and liver failure).

When the Impella^®^ was inserted (pre, during, or post-percutaneous coronary intervention (PCI)) was left to the discretion of the cardiology team in charge of each patient.

Baseline characteristics, clinical, coronary angiography, and echocardiographic data at admission and at 24 h were obtained by reviewing patients’ medical record. LV end-diastolic pressure (LVEDP) elevation was defined in those patients with reduced LV ejection fraction (LVEF) as one or more of the following: E/A ratio > 2, E/Ea ratio ≥ 13 or pulmonary hypertension defined as systolic pulmonary artery pressure ≥ 45 mmHg. Right ventricular (RV) function was assessed based on the tricuspid annular plane systolic excursion (TAPSE) and tricuspid annulus systolic velocity (S’). The cardiac index was calculated using the LV outflow tract diameter, aortic velocity time integral (VTI), heart rate and body surface area. Mitral or aortic regurgitations were classified as moderate to severe according to the first echocardiographic evaluation, based on quantitative, semi-quantitative or qualitative criteria. Right atrial pressure was estimated by the inferior vena cava diameter and its collapsibility. At 24 h, transthoracic echocardiography (TTE) was realized at Impella^®^ level 1 or 2 according to patients’ hemodynamics to evaluate potential myocardial recovery based on previous parameters.

Biological features, especially pH and arterial lactates, renal and liver function, troponin levels, ionogram and blood count, were obtained by computed analyses of patients’ records. Biology at Impella^®^ placement was the closest available before placement. Biology at day one was the closest to 24 h after placement.

Each patient’s prescriptions were reviewed for amine levels at Impella^®^ placement, on day one and on day two, as well as the maximal dose and duration for each drug. We also noted the maximal dose and duration of furosemide therapy during and after Impella^®^ support, fluid challenge, use of amiodarone or levosimendan, as well as any requirement and the duration of non-invasive or invasive ventilation, intra-aortic balloon pump (IABP), venoarterial extracorporeal membrane oxygenation (VA-ECMO) and renal replacement therapy (RRT).

Hemolysis was defined clinically by at least one episode of macroscopic hemoglobinuria (therefore excluding hematuria such as caused by traumatic urinary catheterization), biologically by an elevation of lactate dehydrogenase (>1000 IU/L and unrelated to cytolysis, i.e., despite troponin or transaminases decrease), an elevation of unconjugated bilirubin (>10 µmol/L) and/or an undetectable haptoglobin (inferior to laboratory normal value).

Severe bleeding was defined based on criteria for Bleeding Academic Research Consortium (BARC) stage 3 classification [13]. Blood transfusions (including packed red blood cell, fresh frozen plasma, and platelet concentrates) were counted from Impella^®^ placement to 24 h after withdrawal or during treatment of a local complication such as arterial pseudoaneurysm, using the patients’ transfusion records.

Sepsis was defined as fever, with temperature > 38.5°, and/or a biologic inflammatory syndrome leading to an empiric antibiotic treatment or a modification in an ongoing antibiotic treatment, within 72 h following Impella^®^ placement.

Follow-up was assessed in June 2020 ± 3 months by electronic chart review and/or by phone interview with the patients’ general practitioner/cardiologist, the patient or family for the primary endpoint at one month.

The investigation conforms to the principles outlined in the Declaration of Helsinki. All patients were informed at admission that their clinical data could be used for research purposes according to the principles of the CNIL (French data protection agency) MR-004 methodology and this study was registered under the RnIPH 2020–138.

Based on their 30-day outcome, patients were separated into two groups: patients alive at one month without need for LVAD or heart transplant (recovery group) and patients deceased or alive with LVAD or heart transplant at one month (non-recovery group). 

Continuous variables were expressed as means ± standard deviation or as medians with interquartile ranges (IQR) when not normally distributed. Nominal variables were expressed in numbers and percentages. Association between the mean values of continuous variables was assessed using the Mann–Whitney rank sum test or Student’s t-test when appropriate. Nominal variables were assessed by the χ^2^ test or Fisher’s exact test when appropriate. Regression analysis was performed using variables with a *p*-value < 0.05 and a ratio of 1:10 variables per event, to analyze variables associated with the composite criteria of mortality, LVAD or heart transplants at 30 days, with results reported as odds ratios (OR) with 95% confidence intervals (CI). A *p*-value inferior to 0.05 was considered significant. BiostaTGV^®^ and Stata^®^ (14.2 version) software was used for statistical analyses.

## 3. Results

### 3.1. Population Characteristics 

Sixty-two CS patients with an Impella^®^ support (CP or 5.0) on our unit (Intensive Cardiac Care Unit, Toulouse University Hospital) between January 2011 and January 2020 (Figure 1) were included in our analysis. Furthermore, 49 (79%) and 13 (21%) were, respectively, supported by Impella CP^®^ and Impella 5.0^®^ devices. Their characteristics are summarized in Table 1. They were mainly male (*n* = 55, 89%) with a mean age of 58 ± 11 years. The main CS etiology was acute coronary syndrome (ACS), with or without ST segment elevation (*n* = 57, 92%). The majority of patients had no history of heart failure or coronary artery disease (*n* = 43, 70%).

At Impella^®^ placement, patients presented with severe predominant left ventricular failure (LVEF 22%) with a low cardiac index (1.93 L/min/1.73m^2^), multiorgan failure and inflammatory syndrome. Previous use of vasopressors and inotropes, mechanical ventilation and IABP were frequent. Most patients were directly admitted to the catheterization laboratory (*n* = 54, 87%), with long total ischemic time. Coronarography indicated that the majority of cases were multi-vessel diseases (76%) with total occlusion (TIMI 0 flow) of the culprit artery (65%). 

The majority of the patients were supported by Impella^®^ CP (*n* = 49, 79%), placed by femoral access (*n* = 48, 77%). The majority of Impella^®^ were placed during the first 24 h after admission (*n* = 50, 81%) but mostly after culprit PCI (*n* = 46, 78%), with a median support period of 7 days.

At 24 h, 26% still needed both inotropic and vasopressor support (Table 2). While on Impella^®^ support, 34% of the patients suffered sustained ventricular tachycardia, 25% required RRT for acute kidney injury and 26% needed an escalation to VA-ECMO for persistent hemodynamic compromise or end-organ failure (Table 3). Severe bleedings and limb ischemia were not rare (respectively, in 16 and 13%), with few cases requiring surgical hemostasis (respectively, in 2 and 8%) (Table 4).

### 3.2. 30 Day Outcome

At one month (Figure 1), 27 patients (44%) were alive without requiring LVAD or heart transplant. Within the second group (*n* = 35, 57%), 22 patients died during the first month, 12 were implanted with a LVAD (two of whom died during the first month) and three were transplanted (and alive at one month). At 30 days, survival without requiring LVAD or heart transplantation was not different between Impella CP^®^ and Impella 5.0^®^ supported patients (respectively, 23 (47%) vs. four patients (31%), *p* = 0.30). Moreover, no difference in terms of secondary endpoints was observed (Supplementary Table S1).

### 3.3. Comparative Analysis between Recovery and Non-Recovery Groups at 30 Days

At admission, patients had similar baseline characteristics except for a higher body mass index in the recovery group.

At Impella^®^ placement, patients in the recovery group had a significantly higher blood pressure and higher diuresis (Table 1) but organ dysfunctions and tissue malperfusion were comparable. No difference in terms of LV and RV function was observed, but LVEDP elevation as well as moderate-to-severe mitral regurgitation were more frequent in the non-recovery group (57% vs. 18%; *p* = 0.01 and 38% vs. 15%; *p* = 0.04). Moreover, patients in the non-recovery group required more inotropes and vasopressors. For AMICS, a TIMI 0 flow pre-PCI in the culprit coronary artery was more frequent in the non-recovery group (75% vs. 50%) but primary PCI showed similar results in both groups.

At 24 h, vital signs were comparable between the two groups although a significantly higher LV and RV systolic function were noted in the recovery group (Table 2). There was no significant difference in lactates, troponin peak or acute kidney injury between groups, whereas liver dysfunction was less frequent in the recovery group. At day one, patients in the recovery group required significantly fewer inotropes and vasopressors (dobutamine 7 vs. 44% and noradrenaline 48 vs. 74%). 

They also required, during hospitalization, less invasive mechanical ventilation (56 vs. 80%) and RRT (11 vs. 34%). However, there was no significant difference in duration, maximal power, or maximal flow of Impella^®^, nor in escalation to VA-ECMO (Table 3).

Sepsis and biological hemolysis were frequent especially in the non-recovery group but there was no significant difference in clinical hemolysis, nor in other complications (Table 4).

### 3.4. Factors Associated with 30 Day Outcomes

In univariate analysis, at implantation, mean blood pressure (MBP) (OR 0.96 (0.92–0.99); *p* = 0.04) and inotropic support with dobutamine (OR 3.12 (1.08–8.97); *p* = 0.03) were significantly associated with death, LVAD or heart transplant at 30 days. In multivariate analysis, only inotropic support with dobutamine was associated with our endpoint (OR 3.25 (1.04–10.1); *p* = 0.04) ((Supplementary Table S2).

In univariate analysis, at day one, obesity (OR 0.22 (0.05–0.94); *p* = 0.04), the number of amines (for each amine at day one: OR 4.09 (1.74–9.62); *p* = 0.001), total bilirubin (OR 1.05 (1.00–1.09); *p* = 0.03) and LVEF (for each % increase at day one: OR 0.91 (0.85–0.97); *p* = 0.004) were significantly associated with death, LVAD or heart transplant. Multivariate analysis showed the number of amines (for each amine at day one: OR 4.31 (1.30–14.30); *p* = 0.016), total bilirubin (OR 1.07 (1.00–1.14); *p* = 0.039) and LVEF (for each % increase at day one: OR 0.89 (0.81–0.96); *p* = 0.006) (Table 5).

### 3.5. Factors Associated with 30 Day Outcomes

The median follow-up was 18.7 months (IQR (0.9–34.8)) and six patients (10%) were lost to follow-up. At one year, 24 patients (39%) were alive and had no heart transplant or LVAD, 26 patients (46%) were deceased, 12 (34%) had an LVAD and four (12%) had a transplant. In the recovery group, only two patients (9%) died from non-cardiovascular causes (one hemorrhagic stroke and one septic shock), and the others survived with their native heart.

## 4. Discussion

In our study, which investigated the characteristics of patients with CS treated by percutaneous AFP (Impella^®^ CP or 5.0), the main findings can be summarized as follows: #1. an Impella^®^ device is predominantly used in the case of AMICS in our experience; #2. Impella^®^ appears to be a feasible support for CS patients with hemodynamic compromise and organ hypoperfusion with 44% of the patients surviving without LVAD or heart transplant at 30 days; #3 patients who survived without LVAD or heart transplantation at 30 days experience early myocardial recovery, with LVEF and cardiac output improvements as early as day one after Impella^®^ placement, and #4. patient monitoring and early new evaluation as of day one can highlight factors associated with an unlikely recovery, based on which the heart team can discuss a long-term curative plan for end-stage heart failure.

While the fact that most cases of CS were caused by ACS is consistent with previous CS epidemiologic reports, our study population exhibited some notable differences from the largest series previously described (Supplementary Table S3). We observed a slightly lower mortality rate with 35% of the patients deceased at one month (46% at 1 year), compared to the 37 to 60% in the CARDSHOCK [2], IMP-IT [14] and cVAD [15] registries. Our patients were younger (median age of 58 vs. 64–67 yrs. [2,14,15]) with few having a history of coronary artery disease (18 vs. 66–69% [14,15]) and fewer previous cardiac arrests during initial management (10 vs. 24–40% [2,14,16]). Our patients ranged from stages B to D in the recent Society for Cardiovascular Angiography and Interventions (SCAI) classification, with a majority of patients in stage C (“classic” shock). Even though our patients exhibited higher blood pressure than the lower limits that usually define CS (SBP < 90 mmHg), they presented with genuine clinical or biological signs of systemic hypoperfusion, multi-organ failure and an associated inflammatory syndrome [16]. Furthermore, our patients presented with a less severe and profound shock than patients in the aforementioned registries (less acidosis, lower lactate (lactate 3.1 vs. 4.3–6.1 mmol/L [14,15]) and fewer invasive mechanical ventilations (69 vs. 76–77% [14,15]) even though most required inotropes and/or vasopressors and organ support including mechanical ventilation, RRT, or other circulatory support (prior IABP and/or escalating VA-ECMO). Finally, circulatory support was greater in our study with a more efficient Impella^®^ device: 79% Impella^®^ CP and 21% Impella^®^ 5.0, whereas they were mainly 2.5 in previous registries (57–59% [14,15] vs. 0% in our study).

Patients who survived with their native heart presented with better hemodynamics at implantation, along with less inotropic and vasopressor support, which may indicate quicker management and/or better stabilization prior to Impella^®^ placement. In our population, Impella^®^ was implanted early in the CS course with 19% of the patients without inotropes and/or vasopressors. This is similar to previously published registries that show that early use of Impella^®^ may be associated with an increase in survival in AMICS compared to a classic escalation strategy in refractory CS [17]. This strategy may partially explain our higher survival rate even though few patients in our study were treated by pre-PCI Impella^®^ support, which also seems to be associated with an increase in survival [18,19]. Interestingly, in our specific population of AMICS, with relatively high delays in revascularization, revascularization timing had no impact on short-term outcomes, whereas the severity of the shock and of organ failure was significantly associated with the prognosis. Together, these findings suggest that, in AMICS that occur late after acute coronary occlusion, the most important issue in patient management is not the timing of emergency PCI, as it is in AMI without CS, but rather hemodynamics and end-organ stabilization. Recent clarifications in CS pathophysiology have changed the classic vision of the condition, which has evolved from a static concept of circulatory failure to a dynamic notion of systolic and diastolic LV failure leading to an escalating systemic response (mild-shock phase), resulting in metabolic acidosis and end-organ failure (shock phase) [20]. Early initiation of Impella^®^ could limit this harmful systemic adaptation (including peripheral vasoconstriction and systemic inflammatory response syndrome) and the deleterious effects of inotropic and vasopressor treatment (especially arrhythmias and an increase in myocardial work) and thereby prevent the mild-shock phase from evolving towards actual shock. Further work should be undertaken to better identify prognostic factors of CS progression in AMI, which could lead to early LV unloading as early as in the mild-shock phase, even prior to inotrope/vasopressor use.

Interestingly, after the first evaluation leading to Impella^®^ support, we found that an early new evaluation, as early as day one after Impella^®^ placement, improved the identification of patients for whom failure of AFP weaning was more likely. This should then lead to a discussion by the heart team concerning the initiation of a long-term curative plan for heart failure, namely LVAD or heart transplant. LVEF, a simple parameter, which is reproducible and easy to measure daily at the patient’s bedside, was associated with worse short-term outcomes in multivariate analysis, suggesting that myocardial recovery under LV unloading is a relatively quick process, which should be assessed at an early stage of management. Another important factor was inotropic and/or vasopressor support at day one, which was significantly associated with our endpoint at 30 days, which has already been highlighted at Impella^®^ placement but not after 24 h of Impella^®^ support [18]. Therefore, both the absence of LVEF improvement and the failure of inotrope and/or vasopressor weaning at day one after Impella^®^ support should lead to close monitoring and further evaluation of the feasibility of LVAD or heart transplant, to avoid harmful delays in escalation therapy if needed. Moreover, this strategy appears to accurately predict patient recovery, as 30 day and one-year outcomes were almost identical in our study population. The decision to schedule the implantation of an LVAD or to register the patient on the heart transplant list can only be made in the combined absence of recovery and contraindication detected during the various assessments. This decision will be the subject of a multidisciplinary discussion within the shock team and will be based on the global assessment of every patient.

Our study was observational, retrospective, and monocentric but our series on CS patients with Impella devices is a contribution to the AFP associated literature. Our results reflect the experience and proper practice regarding Impella^®^ use in our center and should be interpreted with caution and confirmed with larger trials, taking into account the heterogeneity of management habits for CS, especially regarding MCS use. MCS indications were discussed through our multidisciplinary CS heart team based on clinical and paraclinical parameters to define the depth of the shock and the severity of the multiple organ failure. However, as in the majority of European centers, we do not have any formal and directive protocols in our center for the management of cardiogenic shock and the MCS implantation (ECMO or Impella^®^), limiting the reproducibility and comparability of our results. Patient data were analyzed retrospectively, and some adverse events may have been underreported, although the Impella-related complication rates observed were consistent with previously published series. The observational nature of our study is also a limitation to the ascertainment of causal relationships, and our results are mainly hypothesis-generating.

Therefore, large-scale prospective studies, which investigate clinical, biological, echocardiographic, and therapeutic features at Impella^®^ placement and at day one after Impella^®^ initiation, are required to confirm the trends we observed in order to improve CS patient management and better define prognostic factors in patients with CS requiring MCS for whom LV recovery will be unlikely. The prospective multicentric randomized trial ULYSS (clinical trial identifier: PHRC-19–0094) organized by the Programme Hospitalier de Recherche Clinique will start soon and may answer these interesting questions.

## 5. Conclusions

LV unloading with Impella^®^ is a growing strategy in CS management. Early Impella^®^ support before profound CS and severe multiorgan failure may improve patient outcomes by hemodynamic and organ stabilization and lead to myocardial recovery. During Impella support, early evaluation, as soon as 24 h after placement, can provide valuable indicators, such as LVEF change or dependence on inotropes/vasopressors, of an unlikely recovery and the need to assess the feasibility of LVAD or heart transplant. Prospective work is needed to better define the appropriate timing for Impella^®^ initiation in CS and prognostic factors of patient recovery with Impella^®^ support.

## Figures and Tables

**Table 1:** Patients’ characteristics at Impella^®^ implantation.

	Cardiogenic Shock (*n* = 62)	Recovery (*n* = 27, 44%)	Non-Recovery (*n* = 35, 56%)	*p*-Value
***Baseline Characteristics***				
Age-yrs.	58 ± 11	58 ± 12	59 ± 10	0.80
Male Gender-no. (%)	55 (88)	24 (89)	31 (89)	0.96
Body Mass Index-kg/m^2^	25.9 ± 4.0	27.1 ± 4.6	25.0 ± 3.3	**0.03**
Current smoker-no. (%)	36 (59)	14 (52)	22 (65)	0.31
Hypertension-no. (%)	22 (36)	10 (37)	12 (35)	0.88
Diabetes mellitus-no. (%)	15 (24)	6 (22)	9 (26)	0.75
Dyslipidemia-no. (%)	17 (27)	8 (30)	9 (26)	0.73
History of heart failure-no. (%)	4 (7)	1 (4)	3 (9)	0.44
History of coronary artery disease-no. (%)	11 (18)	4 (15)	7 (21)	0.56
Peripheral vascular disease-no. (%)	8 (13)	3 (11)	5 (15)	0.71
No history of cardiopathy-no. (%)	42 (70)	20 (77)	22 (65)	0.31
SAPS2	40 ± 19	37 ± 18	43 ± 20	0.23
Cardiac arrest prior to Impella^®^ placement-no. (%)	6 (10)	3 (11)	3 (9)	0.73
***Cardiogenic shock etiology***				
Acute coronary syndrome-no. (%)	57 (91)	26 (96)	31 (88)	0.27
Acute decompensated heart failure-no. (%)	3 (5)	0 (0)	3 (9)	0.12
Acute Myocarditis-no. (%)	1 (2)	1 (4)	0 (0)	0.25
Post-cardiotomy cardiogenic shock-no. (%)	1 (2)	0 (0)	1 (3)	0.38
***Clinical features***				
SBP, mmHg	101 ± 21	108 ± 20	97 ± 21	**0.04**
DBP, mmHg	67 ± 13	70 ± 12	64 ± 13	**0.03**
MBP, mmHg	78 ± 15	83 ± 14	75 ± 15	**0.04**
Heart rate, bpm	107 ± 20	104 ± 16	109 ± 22	0.26
Invasive mechanical ventilation-no. (%)	18 (30)	8 (31)	10 (29)	0.85
Diuresis per hour, liters	0.091 [0.057–0.171]	0.161 [0.078–0.217]	0.079 [0.055–0.107]	**0.01**
***Biology***				
pH	7.37 ± 0.14	7.35 ± 0.14	7.39 ± 0.14	0.36
Lactic acid-mmol/L	3.1 ± 2.1	3.2 ± 2.4	3.0 ± 1.9	0.68
Troponin-ng/L	5030 [471–10776]	6018 [1461–12126]	2547 [269–8671]	0.73
Sodium-mmol/L	136 ± 5	138 ± 3	134 ± 5	**0.004**
Serum creatinine-µmol/L	117 ± 53	107 ± 38	125 ± 61	0.16
GFR-mL/min	68 ± 26	72 ± 26	65 ± 26	0.31
Glycemia-mmol/L	11.8 ± 5.6	11.5 ± 5.6	12.1 ± 5.5	0.66
Total bilirubin-µmol/L	11.2 [7.2–17.7]	12.9 [5.9–15.4]	16.0 [8.0–22.0]	0.31
ASAT-IU/L	180 [72–764]	507 [147–854]	378 [49–662]	0.19
ALAT-IU/L	94 [49–158]	113 [56–160]	121 [46–151]	0.76
GammaGT-IU/L	53 [36–103]	66 [34–84]	85 [40–113]	0.21
Alkaline phosphatase-IU/L	85 [62–103]	90 [59–94]	126 [68–117]	0.15
Hemoglobin-g/dL	13.9 ± 2.3	14.5 ± 2.1	13.4 ± 2.4	0.07
Leukocytes-g/L	17.8 ± 7.1	17.8 ± 7.6	18.0 ± 6.8	0.92
C-reactive proteing/L	67 ± 100	53 ± 96	78 ± 102	0.34
***Echocardiography***				
LVEF-%	22 ± 9	24 ± 10	21 ± 9	0.20
Aortic velocity–time integral-cm	10 ± 4	10 ± 4	9 ± 4	0.43
Cardiac Index-L/min/m^2^	1.93 ± 0.55	1.98 ± 0.61	1.87 ± 0.49	0.69
Elevated LVEDP-no. (%)	16 (42)	3 (18)	13 (57)	**0.01**
Moderate to Severe MR-no. (%)	17 (27)	4 (15)	13 (38)	**0.04**
TAPSE-mm	18 ± 4	19 ± 3	17 ± 5	0.08
***Management***				
Dobutamine-no. (%)	37 (60)	12 (44)	25 (71)	**0.03**
Dobutamine-µg/kg/min	7.61 ± 4.22	5.63 ± 3.05	8.20 ± 4.67	0.06
NAD-no. (%)	41 (66)	15 (56)	26 (74)	0.12
NAD dose-µg/kg/min	0.406 ± 0.479	0.227 ± 0.186	0.527 ± 0.572	**0.03**
Adrenalin-no. (%)	7 (11)	3 (11)	4 (11)	0.97
Adrenalin dose-µg/kg/min	0.423 ± 0.481	0.348 ± 0.501	0.638 ± 0.524	0.53

*p*-Value corresponds to the results of group comparisons using the χ^2^ test or Student’s t/Mann–Whitney test. In bold, *p*-value < 0.05. ALAT: alanine amino-transferase; ASAT: aspartate amino-transferase; DBP: diastolic blood pressure; GFR: glomerular filtration rate; SAPS2: simplified acute physiology score; LVEDP: left ventricular end-diastolic pressure; LVEF: left ventricular ejection fraction; MBP: mean blood pressure; MR: mitral regurgitation; NAD: noradrenalin; SBP: systolic blood pressure; TAPSE: tricuspid annular plane systolic excursion.

**Table 2:** Patients’ characteristics 24 h after Impella^®^ implantation.

	Cardiogenic Shock (*n* = 62)	Recovery (*n* = 27,44%)	Non-Recovery (*n* = 35, 56%)	*p*-Value
***Clinical features***				
SBP, mmHg	99 ± 14	103 ± 14	96 ± 14	0.07
DBP, mmHg	68 ± 9	68 ± 9	67 ± 10	0.54
MBP, mmHg	78 ± 9	80 ± 8	77 ± 9	0.38
Heart rate, bpm	97 ± 18	94 ± 16	100 ± 19	0.26
Invasive mechanical ventilation-no. (%)	35 (58)	13 (50)	22 (65)	0.25
Diuresis per hour, liters	0.073 [0.046–0.102]	0.062 [0.041–0.099]	0.081 [0.065–0.102]	0.24
***Biology***				
pH	7.42 ± 0.08	7.42 ± 0.07	7.42 ± 0.09	0.85
Lactic acid-mmol/L	1.8 [1.2–2.1]	1.6 [1.2–1.8]	2.0 [1.2–2.2]	0.09
Troponin peak-ng/L	20789 [5016–28059]	18444 [5917–24013]	22731 [5016–36142]	0.40
Na-mmol/L	138.3 [135.0–142.0]	139.4 [137.0–142.0]	137.4 [132.0–142.8]	0.13
Creatinine-µmol/L	147 [83–171]	138 [77–142]	153 [89–182]	0.53
GFR-mL/min	62 [35–91]	68 [44–99]	58 [30–82]	0.21
Glycemia-mmol/L	8.7 ± 3.0	8.0 ± 2.1	9.1 ± 3.5	0.14
Total bilirubin-µmol/L	24.9 [14.2–29.0]	19.8 [11.3–25.5]	28.9 [14.9–33.0]	**0.01**
ASAT-IU/L	722 [137–651]	491 [162–590]	910 [112–767]	0.18
ALAT-IU/L	385 [62–176]	185 [57–121]	545 [71–215]	0.13
GammaGT-IU/L	63 [28–80]	44 [24–55]	77 [36–93]	**0.005**
Alkaline phosphatase-IU/L	89 [57–87]	66 [49–73]	109 [62–96]	**0.03**
Hemoglobin-g/dL	11.4 ± 1.8	11.7 ± 1.9	11.1 ± 1.7	0.16
Leukocytes-g/L	16.1 ± 5.8	14.2 ± 5.1	17.5 ± 5.9	**0.02**
C-reactive protein-g/L	141 ± 85	121 ± 94	154 ± 88	0.28
***Echocardiography***				
LVEF-%	22 ± 10.9	27 ± 11.7	18 ± 8.2	**0.001**
Aortic velocity-time integral-cm	10,2 ± 3.7	11.9 ± 3.4	8.9 ± 3.4	**0.004**
TAPSE-mm	18 ± 5.2	19.6 ± 4.8	16.0 ± 5.0	**0.01**
LVEF change *	−0.1 ± 12.2	3.2 ± 14.4	−3.1 ± 9.1	**0.03**
Aortic TVI change *	0.4 ± 4.0	1.4 ± 4.4	−0.9 ± 5.5	**0.04**
TAPSE change *	0.4 ± 5.3	2.1 ± 4.0	−1.2 ± 6.0	0.06
***Management***				
Dobutamine-no. (%)	17 (28)	2 (7)	15 (44)	**0.001**
Dobutamine dose-µg/kg/min	7.38 ± 4.11	10.00 ± 5.00	7.35 ± 3.54	0.29
NAD-no. (%)	38 (62)	13 (48)	25 (74)	**0.04**
NAD dose-µg/kg/min	0.44 ± 0.46	0.26 ± 0.25	0.52 ± 0.52	**0.04**
Adrenalin-no. (%)	4 (7)	0 (0)	4 (11)	0.07
Adrenalin dose-µg/kg/min	1.099 ± 0.864	-	1.099 ± 0.864	-

*p*-value corresponds to the results of group comparisons using the χ^2^ test or the Student’s t-/Mann–Whitney test. In bold, *p*-value < 0.05. ALAT: alanine amino-transferase; ASAT: aspartate amino-transferase; DBP: diastolic blood pressure; GFR: glomerular filtration rate; LVEDP: left ventricular end-diastolic pressure; LVEF: left ventricular ejection fraction; MBP: mean blood pressure; NAD: noradrenalin; SBP: systolic blood pressure; TAPSE: tricuspid annular plane systolic excursion; TVI: time velocity interval. * Change is the difference between the value at admission and 24 h after Impella^®^ placement.

**Table 3:** Cardiogenic shock management.

	Cardiogenic Shock (*n* = 62)	Recovery (*n* = 27, 44%)	Non-Recovery (*n* = 35, 56%)	*p*-Value
Impella^®^ < 24 h after admission- no. (%)	50 (81)	25 (93)	25 (71)	0.036
3.5-no. (%)	49 (79)	23 (85)	26 (74)	0.30
5.0-no. (%)	13 (21)	4 (15)	9 (26)	0.30
Femoral access-no. (%)	48 (77)	23 (85)	25 (71)	0.20
Subclavian access-no. (%)	7 (11)	1 (4)	6 (17)	0.10
Axillar access-no. (%)	7 (11)	3 (11)	4 (11)	0.97
Impella^®^ maximal output-L/min	3.2 ± 0.7	2.9 ± 0.6	3.3 ± 0.8	0.02
Impella^®^ duration-days	6.8 [3.0–8.8]	5.5 [2.0–6.0]	7.9 [4.0–9.5]	0.19
VA-ECMO-no. (%)	16 (26)	5 (19)	11 (31)	0.25
IABP-no. (%)	9 (15)	3 (11)	6 (17)	0.50
Renal replacement therapy-no. (%)	15 (24)	3 (11)	12 (34)	**0.03**
Invasive ventilation-no. (%)	43 (69)	15 (56)	28 (80)	**0.04**
Invasive ventilation median duration-days	8.0 [4.0–11.5]	6.0 [3.0–11.0]	9.0 [4.8–11.3]	0.89
Non-invasive ventilation	24 (39)	9 (33)	15 (43)	0.11
Electric shock-no. (%)	18 (29)	7 (26)	11 (31)	0.64
Dobutamine-no. (%)	49 (79)	20 (74)	29 (83)	0.40
Mean dobutamine support duration-days	3.9 [0.6–4.0]	2.2 [0.1–2]	5.2 [1.0–6.5]	0.02
NAD-no. (%)	53 (86)	21 (78)	32 (91)	0.13
Mean NAD support duration-days	5.4 [1.6–7.8]	3.7 [0.75–3.5]	6.6 [2.8–10.5]	0.08
Adrenalin-no. (%)	16 (26)	6 (22)	10 (29)	0.57
Mean adrenalin support duration-days	0.6 ± 2.0	0.2 ± 0.46	0.9 ± 2.7	0.12
2 or more amines at day one-no. (%)	16 (26)	0 (0)	16 (46)	**<0.001**
Furosemide-no. (%)	56 (90)	24 (89)	32 (91)	0.35
Maximal furosemide dose while on Impella^®^ support-mg per day	273 [0–500]	214 [0–250]	318 [0–500]	0.29
Furosemide duration on Impella^®^ support-days	2.6 [0.0–4.0]	2.1 [0.0–2.5]	2.9 [0.0–4.5]	0.32
Fluid challenge while on Impella^®^ support-Liters	4.9 [2.1–6.4]	4.8 [2.2–5.4]	4.89 [2.1–7.6]	0.94
Levosimendan-no. (%)	10 (16)	3 (11)	7 (20)	0.35
Amiodarone-no. (%)	38 (61)	14 (52)	24 (69)	0.73
***Primary percutaneous coronary intervention (n = 57)***				
Ischemic time > 4 hours-no. (%)	38 (68)	17 (68)	21 (68)	0.98
Multivessel disease-no. (%)	44 (76)	20 (77)	24 (75)	0.86
Pre-PCI Impella^®^ placement-no. (%)	12 (22)	4 (17)	8 (26)	0.46
Culprit PCI-no. (%)	53 (95)	23 (92)	30 (97)	0.43
Immediate complete PCI-no. (%)	23 (41)	11 (44)	12 (39)	0.69
Delayed complete PCI-no. (%)	9 (16)	6 (24)	3 (10)	0.15
Pre-PCI TIMI-no. (%)	37 (65)	13 (50)	24 (75)	**0.046**
post-PCI TIMI 3-no. (%)	50 (88)	21 (84)	28 (90)	0.48

*p*-value corresponds to the results of group comparisons using the χ^2^ test or the Student’s t-/Mann–Whitney test. In bold, *p*-value < 0.05. IABP: intra-aortic balloon pump; NAD: noradrenalin; PCI: percutaneous coronary intervention; VA-ECMO: venoarterial extracorporeal membrane oxygenation.

**Table 4:** Adverse events and one-month outcome.

	Cardiogenic Shock (*n* = 62)	Recovery (*n* = 27, 44%)	Non-Recovery (*n* = 35, 56%)	*p*-Value
***Complications***				
Atrial tachycardia-no. (%)	20 (32)	9 (33)	11 (31)	0.87
Sustained ventricular Tachycardia-no. (%)	21 (34)	7 (26)	14 (40)	0.25
High grade atrioventricular block-no. (%)	5 (8)	0 (0)	5 (14)	**0.04**
Biological hemolysis-no. (%)	54 (89)	19 (73)	35 (100)	**0.001**
Clinical hemolysis-no. (%)	23 (38)	8 (31)	15 (44)	0.29
Sepsis-no. (%)	30 (48)	7 (26)	23 (66)	**0.002**
Severe bleeding-no. (%)	10 (16)	3 (11)	7 (20)	0.35
Impella^®^ access-site bleeding-no. (%)	31 (50)	15 (58)	16 (46)	0.35
Gastrointestinal bleeding-no. (%)	7 (11)	4 (15)	3 (9)	0.67
Hemorrhagic shock-no. (%)	9 (11)	3 (11)	6 (17)	0.72
Bleeding requiring surgical hemostasis-no. (%)	5 (8)	2 (7)	3 (9)	0.87
Bleeding requiring Impella^®^ removal-no. (%)	6 (10)	3 (11)	3 (9)	0.73
Packed red blood cell transfusion	5.2 ± 5.8	4.3 ± 5.7	5.8 ± 5.8	0.32
Fresh frozen plasma transfusion	1.1 ± 2.5	1.0 ± 3.0	1.2 ± 2.1	0.74
Platelet concentrate transfusion	2.5 ± 6.1	2.5 ± 5.4	2.5 ± 6.6	0.99
Limb ischemia-no. (%)	8 (13)	4 (15)	4 (11)	0.72
Limb ischemia requiring vascular surgery-no. (%)	1 (2)	0 (0)	1 (3)	0.36
Ischemic stroke-no. (%)	4 (7)	2 (7)	2 (6)	0.79
Hemorrhagic stroke-no. (%)	0 (0)	0 (0)	0 (0)	-
***Outcome at one month***				
Still hospitalized-no. (%)	23 (37)	9 (33)	14 (40)	0.59
Still hospitalized in ICU-no. (%)	12 (19)	3 (11)	9 (26)	0.15
Hospital readmission-no. (%)	3 (5)	3 (11)	0 (0)	0.08
ICU readmission-no. (%)	2 (3)	2 (7)	0 (0)	0.19
Death-no. (%)	22 (36)	0 (0)	22 (63)	-
LVAD-no. (%)	12 (19)	0 (0)	12 (34)	-
Heart transplant-no. (%)	3 (5)	0 (0)	3 (9)	-

*p*-value corresponds to the results of group comparisons using the χ^2^ test or the Student’s t-/Mann–Whitney test. In bold, *p*-value < 0.05. ICU: intensive care unit; LVAD: left ventricle assist device.

**Table 5:** Results of the univariate and multivariate Cox regression analysis at 24 h to predict the occurrence of mortality, LVAD or heart transplant at 30 days.

	Univariate Analysis		Multivariate Analysis (*n* = 55)	
	OR (95% CI)	*p*-Value	OR (95% CI)	*p*-Value
LVEF (for each more %)	0.91 [0.85–0.97]	**0.004**	0.89 [0.81–0.96]	**0.006**
Number of amines (for each additional amine)	4.09 [1.74–9.62]	**0.001**	4.31 [1.30–14.30]	**0.016**
Total bilirubin	1.05 [1.00–1.09]	**0.03**	1.07 [1.00–1.14]	**0.039**
Obesity (BMI > 30 kg/m^2^)	0.22 [0.05–0.94]	**0.04**	0.16 [0.23–1.08]	0.06

*p*-value corresponds to the results of the Wald test. In bold, *p*-value < 0.05. BMI: body mass index; CI: confidence interval; LVEF: left ventricular ejection fraction; OR: odds ratio.

## Data Availability

The data presented in this study are available on request from the corresponding author.

## Supplementary Materials

The following are available online at https://www.mdpi.com/2077-0383/9/12/4130/s1, Table S1: Thirty-day outcome according to the Impella^®^ device used (Impella CP^®^ versus Impella 5.0^®^). Table S2: Results of the univariate and multivariate Cox regression analysis on admission parameters to predict the occurrence of mortality, LVAD or heart transplant at 30 days. Table S3: Comparison with other Impella^®^ registries.
