# Supplementary material for: Early Evaluation of Patients on Axial Flow Pump Support for Refractory Cardiogenic Shock Is Associated with Left Ventricular Recovery
Source: J Clin Med. 2020 Dec 21;9(12):4130. doi: 10.3390/jcm9124130 (PMC7767477; doi:10.3390/jcm9124130)
Supplement: Supplementary file 1 [file jcm-09-04130-s001.pdf]

Table S1. Thirty-day outcome according to the Impella device used (Impella CP® versus Impella 5.0®).

	**Impella CP (n= 49, 79%)**	**Impella 5.0 (n= 13, 21%)**	**p-value**
	Outcome at one month		
Death - no. (%)	15 (31)	7 (54)	0.12
LVAD - no. (%)	9 (18)	3 (23)	0.70
Heart transplant - no. (%)	3 (6)	0 (0)	0.36

Table S2. Results of the univariate and multivariate Cox regression analysis on admission parameters to predict the occurrence of mortality, LVAD or heart transplant at 30 days.

	Univariate analysis		Multivariate analysis (n=61)	
	OR (95% CI)	P-value	OR (95% CI)	P-value
LVEF (for each additional %)	0.96 [0.91 - 1.02]	0.24	0.95 [0.89 - 1.02]	0.20
MBP (for each additional mmHg)	0.96 [0.92 - 0.99]	**0.04**	0.96 [0.92 - 1.01]	0.09
Dobutamine	3.12 [1.08 - 8.97]	**0.03**	3.25 [1.04 - 10.1]	**0.04**

P-value corresponds to the results of the Wald test**.** In bold, p-value<0.0.5; CI: confidence interval; LVEF: left ventricular ejection fraction; MBP: mean blood pressure; OR: odds ratio.

Table S3. Comparison with other Impella registries.

	**Toulouse (n=62)**	**CARDSHOCK (n= 219)**	**cVAD (n= 287)**	**IMP-IT (n= 229)**
Age – yrs.	58 ± 11	67 ± 12	66	64 ± 13
Male Gender - no. (%)	55 (89)	162 (74)	219 (76)	167 (73)
Body Mass Index - kg/m²	25.9 ± 4.0	26.5 (24.2–29.0)		
History of coronary artery disease - no. (%)	11 (18)	76 (35)	199 (69)	152 (66)
Acute coronary syndrome - no. (%)	57 (92)	177 (81)	287 (100)	172 (75)
SBP at placement, mmHg	101 ± 21	78 ± 14	96 ± 28	
DBP at placement, mmHg	67 ± 13	47 ± 10	58 ± 21	
MBP at placement, mmHg	78 ± 15	57 ± 11	72 ± 22	64 ± 20
Heart rate at placement, bpm	107 ± 20	90 ± 28	93 ± 33	94 ± 24
Sinus rhythm at placement - no. (%)	56 (90)	170 (78)		
Cardiac arrest prior to Impella support	6 (10)	62 (28)	58 (40)	51 (24)
Mechanical ventilation	43 (69)		218 (77)	165 (76)
RRT	15 (24)			62 (27)
LVEF	22 ± 9.0	33 ± 14	25 ± 13	25 ± 12
Cardiac index	1.9 ± 0.6	2.1 ± 0.8		
Moderate to severe MR	17 (27)	73 (35)		
pH	7.37 ± 0.14	7.30 (7.20–7.40)		
Arterial lactates - mmol/l	3.1 ± 2.1	2.8 [1.7–5.8]	4.3 ± 1.1	6.1 ± 4.8
Serum creatinine - µmol/l	117 ± 53	104 (78–140)	150 ± 114	
Total bilirubin - µmol/l	11.2 [7.2-17.7]		20.4 ± 57.8	
Inotrope or vasopressor prior to Impella – no. (%)	50 (81)		230 (80)	
Dobutamine prior to Impella	37 (60)			155 (75)

ECMO	16 (26)	16 (26)		66 (29)
IABP	9 (15)		114 (40)	79 (36)
Pre-PCI Impella	12 (22)		46%	77 (36)
2.5	0 (0)		57%	134 (59)
3.5	49 (79)		42%	84 (37)
5.0	13 (21)		1%	2 (0.8)
Access-site bleeding	31 (50)			25 (11)
Severe bleeding	9 (11)			36 (16)
Limb ischemia	8 (13)			29 (13)
Sepsis	30 (48)			70 (35)

*CARDSHOCK is a global cardiogenic shock registry, does not focus on Impella. DBP: diastolic blood pressure; IABP : intra-aortic balloon pump; oxygenation; LVEF: left ventricle ejection fraction ; MR: mitral regurgitation; MBP: mean blood pressure; PCI: percutaneous coronary intervention; RRT: renal replacement therapy ; SBP: systolic blood pressure; VA-ECMO: veno-arterial extracorporeal membrane oxygenation.
